# Paruresis, Toilet Anxiety, and Gender Minority Victimization Among Transgender Individuals in Urban South India: A Cross-Sectional Study

**DOI:** 10.7759/cureus.115336

**Published:** 2026-08-28

**Authors:** Aswini Lakshmi, R Divya

**Affiliations:** 1 Physiology, Trichy SRM Medical College Hospital and Research Centre, Tiruchirappalli, IND

**Keywords:** bladder health, education and training of medical students and doctors (specialists and phd), paruresis, shy bladder, transgender health

## Abstract

Background

Toilet anxiety and shy bladder syndrome (paruresis) are significant, yet underreported public health concerns among transgender and gender-diverse individuals, who frequently face barriers related to restroom access and safety. Restroom-related anxiety in this population is compounded by social stigma, discrimination, and a lack of safe, inclusive facilities, with downstream effects on urinary behavior, bladder health, and overall well-being.

Objectives

This study aimed to assess toilet anxiety, maladaptive urinary behaviors, bladder health, and victimization experiences among transgender individuals in urban Tamil Nadu, India.

Methods

A cross-sectional observational study was conducted among 50 transgender individuals in the Trichy district of Tamil Nadu, recruited via snowball sampling through community-based networks and non-governmental organizations. Data were collected using structured, self-administered, Tamil-translated and back-translated questionnaires comprising an adapted Urinary Behavior Questionnaire (UBQ), a Bladder Health Questionnaire, an adapted Gender Minority Stress and Resilience (GMSR) victimization module, and an adapted Toilet Anxiety Scale.

Results

The mean participant age was 45.77 ± 7.23 years. Most participants identified as transwomen (n = 38, 76%). More than half belonged to a lower socioeconomic class (n = 26, 52%), and the majority (n = 35, 70%) had undergone some form of medical transition. Maladaptive urinary behavior of moderate-to-severe degree was observed in 27 participants (54%), and moderate-to-severe bladder dysfunction was observed in 26 participants (52%). Gender minority victimization was the most prevalent domain affected, with 36 participants (72%) reporting moderate-to-severe victimization. Moderate-to-severe toilet anxiety was reported by 28 participants (56%), and 28 participants (56%) met criteria for shy bladder syndrome - markedly higher than general-population estimates of 7%-13%.

Conclusions

Transgender individuals in urban South India experience a substantial, multidimensional burden of restroom-related morbidity spanning psychosocial victimization, toilet anxiety, and physiological urinary and bladder dysfunction. These findings underscore the need for inclusive restroom infrastructure, supportive policy enforcement, and transgender-competent clinical training.

## Introduction

Toilet anxiety and shy bladder syndrome (paruresis) are significant yet underreported public health concerns among transgender and gender-diverse individuals, who frequently encounter barriers related to restroom access and safety. Paruresis is defined as the inability or difficulty in urinating in the presence or perceived presence of others and is classified as a form of social anxiety disorder [1-3]. The condition may range in severity from mild hesitation to complete urinary retention and can adversely affect social participation, occupational functioning, and overall quality of life [4].

The prevalence of toilet anxiety in the general population has been estimated to affect up to 20 million individuals worldwide, with reported prevalence rates ranging from 2.8% to 16.4% [1,5]. Previous studies have demonstrated that mild and severe forms of paruresis affect approximately 25.8% and 14.9% of individuals, respectively [6]. Individuals with shy bladder syndrome frequently exhibit avoidance behaviors, including limiting fluid intake, avoiding travel, reducing workplace participation, and withdrawing from social gatherings due to concerns about public restroom use [1,7].

For transgender individuals, restroom-related anxiety is further complicated by social stigma, discrimination, and inadequate access to safe and inclusive facilities. Public restroom access based on gender identity often exposes transgender individuals to harassment, exclusion, and violence [8]. Studies have demonstrated that transgender and gender-diverse individuals frequently avoid public restrooms due to fear of discrimination, which adversely affects both physical and psychological health [9]. Restroom avoidance behaviors may contribute to urinary dysfunction, urinary tract infections, and anxiety disorders [4,10].

Restroom infrastructure and policies play a pivotal role in addressing these challenges. The absence of gender-neutral or single-occupancy facilities, overcrowding, and inadequate privacy have been associated with elevated levels of anxiety and paruresis [5]. Previous negative experiences, bullying, and shame associated with restroom use have also been identified as significant contributing factors [6,10]. Among transgender individuals, gender dysphoria and social marginalization may further intensify these experiences [11,12].

The psychological burden associated with restroom-related anxiety in this population is substantial. Such experiences may impair access to education, employment, and social participation, thereby contributing to social isolation and a diminished quality of life [8]. Chronic restroom-related anxiety may also predispose individuals to depression, low self-esteem, and social withdrawal [7,9].

This study aimed to assess toilet anxiety, urinary behaviors, bladder health, and victimization experiences among transgender individuals in urban Tamil Nadu.

## Materials and methods

Study design and setting

A cross-sectional observational study was conducted in the Trichy district of Tamil Nadu, India.

Study population

The study recruited transgender individuals aged 18 years and above, in collaboration with local non-governmental organizations working with transgender communities in urban Tamil Nadu.

Sample size

The sample size was calculated based on a prior study by Herman, which reported a 68% prevalence of verbal harassment among transgender individuals [10]. Using a confidence level of 95% and a relative precision of 20%, the estimated sample size was derived using the following formula: $n = \frac{Z^2 \times p \times (1-p)}{d^2}$, where Z = 1.96, p = 0.68, and d = 0.136. The calculated and adopted sample size was 50 participants.

Sampling technique

Participants were recruited using a snowball sampling method through community-based networks and non-governmental organizations operating within the Trichy district.

Data collection tools

Structured, self-administered questionnaires were used to collect both quantitative and qualitative data (Appendix 1). Instruments included adapted Toilet Anxiety Scale modules, Bladder Health Questionnaire modules, and open-ended sections for participant reflections. All questionnaires were translated into Tamil and back-translated to ensure linguistic validity and comprehension [7,10,13-16]. All questionnaires and scales utilized in this study (Urinary Behavior Questionnaire (UBQ), Bladder Health Questionnaire, Gender Minority Stress and Resilience (GMSR)-adapted module, and Toilet Anxiety Scale) are open for non-commercial research use and were properly cited in accordance with the original authors' guidelines; no formal licenses were required.

Statistical analysis

Data were analyzed using IBM SPSS Statistics for Windows, Version 29 (Released 2022; IBM Corp., Armonk, NY, USA). Descriptive statistics, including frequencies, percentages, means, and standard deviations, were calculated.

Ethical considerations

Institutional ethical approval was obtained prior to the commencement of the study (IRB reference number: LXXIX-IRB-01, Trichy SRM IEC - Institutional Ethics Committee). Written informed consent was obtained from all participants, and confidentiality and anonymity were maintained throughout the study.

## Results

Fifty transgender individuals participated in this study. The sociodemographic profile of all participants is presented in Table 1.

**Table 1:** Sociodemographic Characteristics of Study Participants (N = 50) Medical transition refers to hormonal therapy and/or gender-affirming surgical procedures undertaken prior to study enrollment. Socioeconomic classification was determined based on participant-reported monthly household income and primary occupation. Individual subcategory percentages may not total 100% due to rounding. SD: standard deviation

Variable	Category	n	%
Age (Mean ± SD)	45.77 ± 7.23 years	-	-
Gender Identity	Transwoman	38	76
	Transman	7	14
	Non-binary	4	8
	Others	1	2
Socioeconomic Class	Upper	3	6
	Middle	21	42
	Lower	26	52
Medical Transition Status	Yes	35	70
	No	15	30

The mean age was 45.77 ± 7.23 years. The majority of participants identified as transwomen (n = 38, 76%), followed by transmen (n = 7, 14%), non-binary individuals (n = 4, 8%), and others (n = 1, 2%). With regard to socioeconomic status, n = 26 (52%) belonged to the lower class, n = 21 (42%) to the middle class, and n = 3 (6%) to the upper class, indicating that more than half of the participants were from economically disadvantaged backgrounds. The majority of participants (n = 35, 70%) reported having undergone some form of medical transition, encompassing hormonal therapy and/or gender-affirming surgical procedures, while n = 15 (30%) had not undergone any medical intervention at the time of the study.

Urinary behavior

Urinary behavior was assessed using the UBQ, a self-reported, eight-item instrument scored on a Likert scale of 0 (never) to 4 (often), with a total possible score range of 0-32. Higher scores indicate a greater frequency of maladaptive urinary behaviors. Item-level scores and categorical distribution are presented in Tables 2-3.

**Table 2:** Urinary Behavior Questionnaire - Item Mean Scores (N = 50) Each item was scored on a Likert scale: 0 = never, 1 = rarely, 2 = sometimes, 3 = often, and 4 = very often. The total possible score range per item was 0-4. The overall mean score was calculated as the mean of all eight item means. Items were presented in descending order of mean score [7,13]. SD: standard deviation; UBQ: Urinary Behavior Questionnaire

Item	Mean ± SD
I delay urination even when I feel the urge	3.04 ± 0.97
I reduce fluid intake to avoid needing to urinate in public	2.88 ± 1.03
I use the restroom "just in case" before leaving a safe space	2.76 ± 1.03
I experience anxiety while searching for a restroom	2.72 ± 1.30
I avoid drinking before social events to prevent restroom use	2.58 ± 1.12
I avoid using unfamiliar restrooms	2.52 ± 1.20
I plan my route or daily activities around toilet access	2.26 ± 1.31
I use specific coping strategies to manage public toilet use	2.08 ± 1.37
Overall Mean Score	2.61 ± 1.1

**Table 3:** Score Category Distribution Across All Instruments (N = 50) Consolidated score category distributions for all four instruments assessed in the study. UBQ (Urinary Behavior Questionnaire) total possible score range: 0-32; Bladder Health Questionnaire total possible score range: 0-32; Gender Minority Victimization Scale total possible score range: 0-45; Toilet Anxiety Scale total possible score range: 0-32. Score cutoffs: UBQ: No = 0-8, Mild = 9-16, Moderate = 17-24, Severe = >24; Bladder Health: No = 0-8, Mild = 9-16, Moderate = 17-24, Severe = >24; Victimization: Low = <18, Mild = 18-27, Moderate = 28-36, Severe = >36; Toilet Anxiety: No = <8, Mild = 8-16, Moderate = 17-23, Severe = ≥24. Percentages may not total 100% due to rounding [7,10,13-16].

Instrument/Domain	Score Category	Score Range	n (Frequency)	Percentage (%)
Urinary Behavior Questionnaire	No unhealthy behavior	0-8	7	14%
	Mild unhealthy behavior	9-16	16	32%
	Moderate unhealthy behavior	17-24	17	34%
	Severe unhealthy behavior	>24	10	20%
Bladder Health Questionnaire	No bladder problems	0-8	6	12%
	Mild problems	9-16	18	36%
	Moderate problems	17-24	19	38%
	Severe problems	>24	7	14%
Gender Minority Victimization Scale	Low	<18	4	8%
	Mild	18-27	10	20%
	Moderate	28-36	25	50%
	Severe	>36	11	22%
Toilet Anxiety Scale	No anxiety	<8	8	16%
	Mild anxiety	8-16	14	28%
	Moderate anxiety	17-23	18	36%
	Severe anxiety	≥24	10	20%

The overall mean UBQ score was 2.61 ± 1.1, indicating a moderate-to-high level of maladaptive urinary behavior across the study population. Among individual items, the highest mean score was recorded for delayed urination despite feeling the urge to void (3.04 ± 0.97), followed by restriction of fluid intake to avoid the need to urinate in public (2.88 ± 1.03) and pre-emptive restroom use before leaving a perceived safe space (2.76 ± 1.03). Anxiety while searching for a restroom (2.72 ± 1.30) and avoidance of drinking before social events (2.58 ± 1.12) also yielded notably elevated scores. The lowest-scoring item was the use of specific coping strategies to manage public toilet use (2.08 ± 1.37); however, this value still falls within the moderate range on the adopted scale. The uniformly elevated scores across all eight items - with no item falling below 2.0 - indicate a pervasive and consistent pattern of behavioral adaptation to restroom-related challenges within this population.

Collectively, n = 27 (54%) of participants demonstrated moderate-to-severe maladaptive urinary behavior, highlighting a substantial burden of restroom-driven behavioral modification in this study population.

Bladder health

Bladder health was assessed using a validated, eight-item Bladder Health Questionnaire scored on a Likert scale of 0 (never) to 4 (often), with a total possible score range of 0-32. Higher scores reflect a greater frequency or severity of bladder symptoms. Categorical distribution and item-level scores are presented in Table 3 and Table 4.

**Table 4:** Bladder Health Questionnaire - Item Mean Scores (N = 50) Each item was scored on a Likert scale: 0 = never, 1 = rarely, 2 = sometimes, 3 = often, and 4 = very often. The total possible score range per item was 0-4. The overall mean score was calculated as the mean of all eight item means. Items were presented in descending order of mean score [14]. SD: standard deviation

Item	Mean ± SD
I needed to urinate more frequently than usual	2.56 ± 1.08
I have experienced urgency to urinate	2.52 ± 1.12
I have avoided restrooms due to safety concerns or gender-based fears	2.48 ± 1.13
My bladder issues have interfered with my daily activities	2.48 ± 1.09
I had pain or burning during urination	2.46 ± 1.24
I leaked urine when I did not want to	2.44 ± 1.16
I felt that I could not completely empty my bladder	2.42 ± 1.05
I woke up during the night to urinate	2.42 ± 1.14
Overall Mean Score	2.46 ± 1.11

The overall mean bladder health score was 2.46 ± 1.11, indicating a moderate degree of lower urinary tract dysfunction across the study population. Among individual items, the highest mean scores were recorded for increased urinary frequency (2.56 ± 1.08) and urgency (2.52 ± 1.12), suggesting these as the most prevalent lower urinary tract symptoms in this cohort. Restroom avoidance due to safety concerns or gender-based fears (2.48 ± 1.13) and interference of bladder issues with daily activities (2.48 ± 1.09) were also prominently elevated, indicating that urinary dysfunction was not merely physiological but substantially influenced by social and environmental factors. Dysuria was reported with a mean score of 2.46 ± 1.24. Urinary incontinence (2.44 ± 1.16), incomplete bladder emptying (2.42 ± 1.05), and nocturia (2.42 ± 1.14) recorded the lowest mean scores, though all remained within the moderate range. The consistent scoring pattern across all items reflects a clinically meaningful and multifaceted burden of lower urinary tract dysfunction in this population. Collectively, n = 26 (52%) of participants demonstrated moderate-to-severe bladder dysfunction, representing a considerable health burden among study participants.

Gender minority victimization

Gender minority victimization was assessed using nine items adapted from the GMSR measure, reflecting the frequency of discriminatory, harassing, and harmful experiences encountered by participants. Categorical distribution and item-level scores are summarized in Table 3 and Table 5.

**Table 5:** Gender Minority Victimization Scale - Item Mean Scores (N = 50) Items were adapted from Testa et al. (2015). Each item was scored on a Likert scale reflecting frequency: 0 = never, 1 = rarely, 2 = sometimes, 3 = often, and 4 = very often. The total possible score range per item was 0-4. The overall mean score was calculated as the mean of all nine item means. Items were presented in descending order of mean score [15]. SD: standard deviation; GMSR: Gender Minority Stress and Resilience Measure

Item	Mean ± SD
Avoidance due to fear	3.82 ± 1.02
Verbal harassment	3.80 ± 1.00
Unsafe in restrooms	3.74 ± 1.04
Unwanted sexual contact	3.72 ± 1.05
Non-consensual touch/fetishization	3.70 ± 1.06
Physical threat/injury	3.68 ± 1.05
Sexual harassment	3.66 ± 1.01
Threats (online/in-person)	3.66 ± 1.03
Denied services/mistreated	3.62 ± 1.07
Overall Mean Score	3.71 ± 1.04

The overall mean victimization score was 3.71 ± 1.04, indicating markedly elevated and near-constant levels of discrimination and harassment across the study population. Among the nine items, avoidance due to fear recorded the highest mean score (3.82 ± 1.02), closely followed by verbal harassment (3.80 ± 1.00), safety concerns in public restrooms (3.74 ± 1.04), and unwanted sexual contact (3.72 ± 1.05). Non-consensual touch and fetishization (3.70 ± 1.06), physical threat or injury (3.68 ± 1.05), sexual harassment (3.66 ± 1.01), and threats - online or in-person (3.66 ± 1.03) - also yielded critically elevated scores. The lowest-scoring item was denial of services or mistreatment (3.62 ± 1.07); however, even this value approaches the maximum on the adopted scale. Notably, the inter-item range across all nine victimization items was only 0.20 points (3.62-3.82), indicating a uniformly severe and pervasive pattern of victimization with minimal variance between item types. This suggests that participants experienced multiple forms of discrimination simultaneously and habitually, rather than selectively. Collectively, n = 36 (72%) of participants experienced moderate-to-severe gender minority victimization - the highest proportion of any domain measured in this study - underscoring the deeply entrenched and systemic nature of discrimination faced by this population.

Toilet anxiety

Toilet anxiety was assessed using an eight-item adapted Toilet Anxiety Scale scored from 0 (never) to 4 (often), with a total possible score of 0-32. Higher scores denote greater levels of restroom-related anxiety. Categorical distribution and item-level scores are presented in Table 3 and Table 6.

**Table 6:** Toilet Anxiety Scale - Item Mean Scores (N = 50) Each item was scored on a Likert scale: 0 = never, 1 = rarely, 2 = sometimes, 3 = often, and 4 = very often. The total possible score range per item was 0-4. The overall mean score was calculated as the mean of all eight item means. Items were presented in descending order of mean score [10,16]. SD: standard deviation

Item	Mean ± SD
I feel anxious when I need to use a public restroom	2.94 ± 0.98
I feel unsafe using restrooms due to my gender identity	2.92 ± 0.95
I feel uncomfortable due to lack of privacy in restrooms	2.73 ± 0.96
I feel stressed about restroom access when planning outings	2.59 ± 1.03
I avoid using public restrooms whenever possible	2.56 ± 0.99
I find it difficult to urinate when others are nearby	2.52 ± 1.01
I worry about being judged while using a restroom	2.37 ± 1.05
I delay urination because I cannot find a safe restroom	2.30 ± 0.99
Overall Mean Score	2.67 ± 1.11

The overall mean toilet anxiety score was 2.67 ± 1.11, indicating moderate-to-high levels of restroom-related anxiety across the study population. The highest item score was recorded for feeling anxious when needing to use a public restroom (2.94 ± 0.98), closely followed by feeling unsafe in restrooms due to gender identity (2.92 ± 0.95). These two items - both directly rooted in identity-based safety concerns - yielded the highest scores, suggesting that perceived personal risk, rather than physiological difficulty alone, is the dominant driver of toilet anxiety in this population. Discomfort due to lack of restroom privacy (2.73 ± 0.96), stress related to restroom access during outings (2.59 ± 1.03), and avoidance of public restrooms wherever possible (2.56 ± 0.99) also yielded notably elevated scores. The lowest-scoring item was delay of urination due to inability to locate a safe restroom (2.30 ± 0.99), which nonetheless reflects a moderate and clinically significant level of anxiety on the adopted scale. Taken together, the item-level pattern indicates that safety- and identity-related concerns are the primary contributors to toilet anxiety, with practical access barriers serving as a secondary amplifying factor.

Collectively, n = 28 (56%) of participants experienced moderate-to-severe toilet anxiety.

Shy bladder syndrome prevalence

Shy bladder syndrome (paruresis) was assessed based on participant responses to items reflecting difficulty initiating urination in social or public settings. Nearly n = 28 (56%) of transgender participants met the criteria for shy bladder syndrome, while n = 22 (44%) did not.

Summary of key findings

Across all four domains assessed, the majority of study participants demonstrated clinically significant burden. The combined prevalence of moderate-to-severe outcomes was as follows: gender minority victimization, n = 36 (72%); shy bladder syndrome, n = 28 (56%); toilet anxiety, n = 28 (56%); maladaptive urinary behaviors, n = 27 (54%); and bladder dysfunction, n = 26 (52%). These findings collectively indicate a high, multidimensional, and mutually reinforcing burden of restroom-related morbidity among transgender individuals in urban South India, rooted in both psychosocial and physiological domains.

## Discussion

This study examined urinary behaviors, bladder health, victimization experiences, and toilet-related anxiety in transgender individuals in urban Tamil Nadu, revealing a significant and clinically meaningful burden of both physical symptoms and psychosocial distress. The mean age of participants (45.77 ± 7.23 years) reflects a relatively older demographic than is typically represented in transgender health research, where younger individuals are frequently overrepresented. Furthermore, the majority of participants identified as transwomen (n = 38, 76%), a finding consistent with community-based research from India, which indicates greater visibility and community participation among transwomen relative to transmen [12].

The UBQ results revealed an overall mean score of 2.61 ± 1.1, suggesting a moderate-to-high prevalence of maladaptive urinary behaviors. Delayed urination and fluid restriction exhibited the highest mean values, reflecting behavioral adaptations aimed at circumventing unsafe or inaccessible restroom environments. These observations are consistent with findings by Reynolds et al., in which approximately 47% of participants reported postponing voiding and 39% reported fluid restriction - both behaviors significantly correlated with exacerbation of lower urinary tract symptoms [7]. Notably, shy bladder syndrome in the general population has been estimated to affect 7%-13% of individuals [5,6], with higher prevalence observed in socially stressful contexts. In the present study, shy bladder syndrome was identified in n = 28 (56%) of the transgender participants - a substantially higher prevalence than general-population estimates. This marked disparity underscores the profound impact of social marginalization and minority stress on urinary function in this community.

In this study, n = 17 (34%) of participants exhibited moderate unhealthy urinary behavior, and n = 10 (20%) exhibited severe unhealthy behavior, suggesting a heightened burden attributable to compounded minority stress. The bladder health data corroborated this trend, with a mean score of 2.46 ± 1.11 indicating a moderate degree of bladder dysfunction. Urinary frequency, urgency, and dysuria were the most commonly reported symptoms. These results align with those of Coyne et al., who found that more than 45% of adults experienced at least one lower urinary tract symptom, which was associated with significantly diminished quality of life [14]. Additionally, Bradley et al. demonstrated that urinary problems such as incontinence and incomplete bladder emptying substantially interfere with daily functioning [13]. In the present study, n = 19 (38%) of participants reported moderate bladder problems and n = 7 (14%) reported severe problems - rates notably higher than those typically reported in the general population - suggesting heightened vulnerability among transgender individuals.

The most clinically significant findings pertained to gender minority victimization, with an overall mean score of 3.71 ± 1.04 indicating markedly elevated levels of discrimination and harassment. Verbal harassment, avoidance driven by fear, and safety concerns in public restrooms were the most prominent contributors. These findings are consistent with the seminal work of Herman, which demonstrated that 68% of transgender individuals had experienced verbal harassment and 9% had been physically assaulted in restroom settings [12]. Furthermore, Testa et al. established that minority stress significantly exacerbates adverse mental and physical health outcomes for transgender individuals [15]. In the present study, n = 25 (50%) of participants reported moderate victimization and n = 11 (22%) experienced severe victimization, highlighting the pervasive nature of discrimination faced by this population.

Toilet anxiety scores were also elevated, with a mean of 2.67 ± 1.11, reflecting moderate-to-high levels of anxiety. Lack of privacy, fear of judgment, and safety concerns in public restrooms emerged as primary drivers. These findings are consistent with the broader literature on paruresis and toilet anxiety, which estimates that approximately 25%-30% of individuals experience significant anxiety when using public restrooms [1,4]. Furthermore, Hutchings and Kehinde found that approximately 20% of individuals with paruresis experience severe functional impairment, including pronounced avoidance behaviors [5,6]. Bagagli et al. observed that more than 60% of transwomen refrained from using public restrooms due to concerns about discrimination, adversely affecting both their health and self-worth [8]. Okoli et al. reported that approximately 70% of transgender individuals avoided restrooms, a behavior significantly correlated with both anxiety and urinary dysfunction [9]. The current study corroborates these findings, with n = 18 (36%) of participants experiencing moderate toilet anxiety and n = 10 (20%) reporting severe anxiety.

Collectively, these findings indicate a disproportionately high burden of victimization, toilet anxiety, and maladaptive urinary behaviors in this study population, underscoring the critical need for inclusive restroom policies, improved infrastructure, and targeted health interventions.

Limitations

The primary limitations of this study include the use of non-probability snowball sampling, which may limit generalizability to the broader transgender population, and the potential for underreporting of abuse-related experiences due to shame or fear among participants. Additionally, the relatively small sample size (N = 50) warrants careful contextual consideration. While this cohort size is modest compared to larger general-population prevalence studies examining shy bladder syndrome (many of which include several thousand participants) [5,6], the sample is appropriate for this cross-sectional descriptive study of a hard-to-reach, marginalized population in a specific geographic region. Transgender individuals in urban South India represent a particularly vulnerable and socially isolated subpopulation with limited accessibility through standard sampling frames; consequently, the snowball sampling methodology and resulting sample size reflect the practical realities of community-based research in this context. This study should therefore be understood as providing rich, localized descriptive evidence of restroom-related morbidity in this specific urban population rather than as a nationally representative epidemiological estimate. Future investigations employing larger, geographically diverse samples and prospective longitudinal designs will be essential to evaluate the generalizability and temporal trajectory of these findings across broader transgender communities in India.

## Conclusions

This study highlights the substantial and interconnected burden of restroom-related challenges experienced by transgender individuals in urban South India, spanning gender minority victimization, toilet anxiety, maladaptive urinary behaviors, shy bladder syndrome, and clinically meaningful bladder dysfunction. These outcomes are not isolated morbidities but appear to form a cascade of harm, in which pervasive safety concerns, social stigma, and structural barriers to inclusive restroom access drive behavioral adaptation, which in turn contributes to physiological dysfunction and psychological distress.

Addressing this burden requires coordinated action across multiple levels: provision of gender-neutral, private restroom infrastructure in public spaces, healthcare institutions, workplaces, and educational settings; active enforcement of inclusive restroom access policies consistent with existing transgender rights legislation; training of healthcare providers - particularly in primary care, urology, and psychiatry - to screen for and manage restroom-related urinary dysfunction and toilet anxiety in transgender patients; and integration of psychosocial interventions such as cognitive behavioral therapy and peer support into transgender health services. Incorporating transgender health and physiology into medical curricula is similarly essential to equip future clinicians to deliver affirming, competent care. Future research employing longitudinal designs, larger and more geographically diverse samples, and culturally validated instruments is warranted to further characterize this burden and to evaluate the impact of inclusive infrastructure and supportive policy on transgender health outcomes. Ultimately, restroom access should be recognized as a determinant of physical health, psychological well-being, occupational participation, and social dignity for transgender individuals, requiring sustained, coordinated effort from healthcare providers, medical educators, policymakers, urban planners, and civil society alike.

## Appendices

Appendix 1. Study assessment battery & scoring protocols

Research Article: Paruresis, Toilet Anxiety, and Gender Minority Victimization Among Transgender Individuals in Urban South India: A Cross-Sectional Study

SECTION A: TOILET ANXIETY SCALE (Adapted TAS)

Assessment of anxiety and discomfort experienced when using public restrooms, particularly related to gender identity and safety concerns.

Response Scale:

0 = Never  |  1 = Rarely  |  2 = Sometimes  |  3 = Often  |  4 = Very Often

Items (8 items; Scoring Range: 0-32)

1. I feel anxious when I need to use a public restroom

2. I feel unsafe using restrooms due to my gender identity

3. I feel uncomfortable due to lack of privacy in restrooms

4. I feel stressed about restroom access when planning outings

5. I avoid using public restrooms whenever possible

6. I find it difficult to urinate when others are nearby

7. I worry about being judged while using a restroom

8. I delay urination because I cannot find a safe restroom

Scoring Protocol:

Total Score = Sum of all 8 items (Score Range: 0-32)

Category Cutoffs:

(a) No anxiety: <8

(b) Mild anxiety: 8-16

(c) Moderate anxiety: 17-23

(d) Severe anxiety: ≥24

SECTION B: GENDER MINORITY VICTIMIZATION SCALE (Adapted GMVS)

Measurement of experiences of discrimination, harassment, and victimization related to gender minority status. Adapted from Testa et al. (2015) Gender Minority Stress and Resilience Measure (GMSR).

Response Scale:

0 = Never  |  1 = Rarely  |  2 = Sometimes  |  3 = Often  |  4 = Very Often

Items (9 items; Scoring Range: 0-45)

1. Avoidance due to fear

2. Verbal harassment

3. Unsafe in restrooms

4. Unwanted sexual contact

5. Non-consensual touch/fetishization

6. Physical threat/injury

7. Sexual harassment

8. Threats (online/in-person)

9. Denied services/mistreated

Scoring Protocol:

Total Score = Sum of all 9 items (Score Range: 0-45)

Category Cutoffs:

(a) Low: <18

(b) Mild: 18-27

(c) Moderate: 28-36

(d) Severe: >36

SECTION C: BLADDER HEALTH QUESTIONNAIRE (Adapted BHQ)

Assessment of urinary symptoms and lower urinary tract dysfunctions experienced during daily life.

Response Scale:

0 = Never  |  1 = Rarely  |  2 = Sometimes  |  3 = Often  |  4 = Very Often

Items (8 items; Scoring Range: 0-32)

1. I have needed to urinate more frequently than usual

2. I have experienced urgency to urinate

3. I have avoided restrooms due to safety concerns or gender-based fears

4. My bladder issues have interfered with my daily activities

5. I have experienced pain or burning during urination

6. I have leaked urine when I did not want to

7. I have felt that I could not completely empty my bladder

8. I have woken up during the night to urinate

Scoring Protocol:

Total Score = Sum of all 8 items (Score Range: 0-32)

Category Cutoffs:

(a) No bladder problems: 0-8

(b) Mild problems: 9-16

(c) Moderate problems: 17-24

(d) Severe problems: >24

SECTION D: URINARY BEHAVIOR QUESTIONNAIRE (UBQ)

Measurement of behavioral adaptations and coping strategies related to urinary function and restroom access in social settings.

Response Scale:

0 = Never  |  1 = Rarely  |  2 = Sometimes  |  3 = Often  |  4 = Very Often

Items (8 items; Scoring Range: 0-32)

1. I delay urination even when I feel the urge

2. I reduce fluid intake to avoid needing to urinate in public

3. I use the restroom "just in case" before leaving a safe space

4. I experience anxiety while searching for a restroom

5. I avoid drinking before social events to prevent restroom use

6. I avoid using unfamiliar restrooms

7. I plan my route or daily activities around toilet access

8. I use specific coping strategies to manage public toilet use

Scoring Protocol:

Total Score = Sum of all 8 items (Score Range: 0-32)

Category Cutoffs:

(a) No unhealthy behavior: 0-8

(b) Mild unhealthy behavior: 9-16

(c) Moderate unhealthy behavior: 17-24

(d) Severe unhealthy behavior: >24

VERIFICATION & ALIGNMENT NOTES:

All instruments in this battery were adapted and translated into Tamil for administration to a sample of transgender individuals in urban South India (N = 50). Instruments were administered as self-report questionnaires in the language preferred by participants. Data were analysed using IBM SPSS Statistics Version 29. Descriptive statistics and category distributions are reported in the main manuscript. The scoring category cutoffs presented above align with Table 3 (Score Category Distribution Across All Instruments, N = 50) in the results section of the manuscript. For questions regarding these instruments or the scoring protocols, please contact the corresponding author.
